# ChEBI: re-engineered for a sustainable future

**DOI:** 10.1093/nar/gkaf1271

**Published:** 2025-11-28

**Authors:** Adnan Malik, Muhammad Arsalan, Carlos Moreno, Juan Mosquera, Eloy Félix, Tevfik Kizilören, Venkatesh Muthukrishnan, Barbara Zdrazil, Andrew R Leach, Noel M O’Boyle

**Affiliations:** European Molecular Biology Laboratory, European Bioinformatics Institute (EMBL-EBI), Wellcome Genome Campus, Hinxton, Cambridgeshire CB10 1SD, United Kingdom; European Molecular Biology Laboratory, European Bioinformatics Institute (EMBL-EBI), Wellcome Genome Campus, Hinxton, Cambridgeshire CB10 1SD, United Kingdom; European Molecular Biology Laboratory, European Bioinformatics Institute (EMBL-EBI), Wellcome Genome Campus, Hinxton, Cambridgeshire CB10 1SD, United Kingdom; European Molecular Biology Laboratory, European Bioinformatics Institute (EMBL-EBI), Wellcome Genome Campus, Hinxton, Cambridgeshire CB10 1SD, United Kingdom; European Molecular Biology Laboratory, European Bioinformatics Institute (EMBL-EBI), Wellcome Genome Campus, Hinxton, Cambridgeshire CB10 1SD, United Kingdom; European Molecular Biology Laboratory, European Bioinformatics Institute (EMBL-EBI), Wellcome Genome Campus, Hinxton, Cambridgeshire CB10 1SD, United Kingdom; European Molecular Biology Laboratory, European Bioinformatics Institute (EMBL-EBI), Wellcome Genome Campus, Hinxton, Cambridgeshire CB10 1SD, United Kingdom; European Molecular Biology Laboratory, European Bioinformatics Institute (EMBL-EBI), Wellcome Genome Campus, Hinxton, Cambridgeshire CB10 1SD, United Kingdom; European Molecular Biology Laboratory, European Bioinformatics Institute (EMBL-EBI), Wellcome Genome Campus, Hinxton, Cambridgeshire CB10 1SD, United Kingdom; European Molecular Biology Laboratory, European Bioinformatics Institute (EMBL-EBI), Wellcome Genome Campus, Hinxton, Cambridgeshire CB10 1SD, United Kingdom

## Abstract

Chemical Entities of Biological Interest (ChEBI) is a high-quality, manually curated, and open-access database and ontology of chemical entities available online at https://www.ebi.ac.uk/chebi/. The chemical entities in question are either naturally occurring compounds or synthetic compounds that play a vital role in the processes of living organisms. ChEBI was launched in 2004, and over the years the original codebase has become increasingly difficult to maintain. Here, we describe the complete overhaul and modernization of ChEBI’s infrastructure, including its codebase and associated tools (website, web services, and submission tool) to ensure the continued availability and growth of this critical resource for the global bioinformatics community and beyond. The infrastructure overhaul also enabled us to introduce new features and capabilities into ChEBI as well as to update or deprecate redundant ones.

## Graphical Abstract

**Graphical Abstract:**
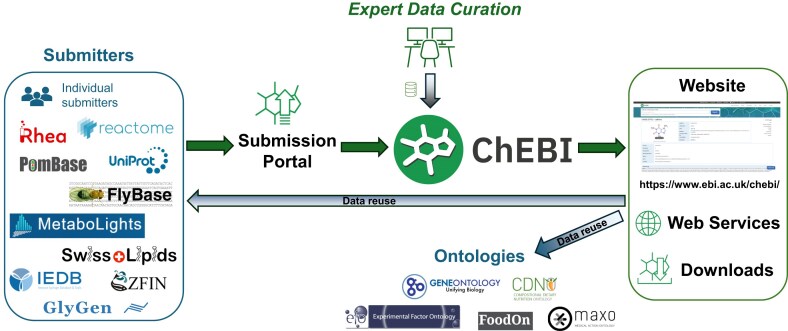

## Introduction

Chemical entities ranging from atoms to the most complex molecules are fundamental to life and are essential components of all living organisms [1]. They are therefore of significant interest to the scientific community. In the scientific literature and in databases, chemical entities are frequently referenced by multiple names. To take one very simple example, the non-steroidal anti-inflammatory drug aspirin is referred to as acetylsalicylic acid, 2-(acetyloxy)benzoic acid, and *o*-acetylsalicylic acid among many other synonyms. This ambiguity and complexity can lead to confusion, errors, misleading conclusions, and potentially fatal consequences [2–4]. ChEBI acts as a reliable and trusted resource that provides ‘definitive’ information about chemical entities, thereby delivering a solution to many of these challenges. ChEBI incorporates standard naming systems from global bodies such as the International Union of Pure and Applied Chemistry (IUPAC; https://iupac.org/) and the Nomenclature Committee of the International Union of Biochemistry and Molecular Biology (https://iubmb.org/). The database also provides chemical, biological, and semantic information [5]. Critically, ChEBI is structurally enabled—it contains a precise computer representation of the molecular structure, which enables users to undertake multiple types of structural queries (such as substructure and similarity searches).

ChEBI creates for each distinct molecular structure a unique and stable identifier (ChEBI ID), which is used by multiple other data resources to definitively identify that specific compound [5], much as a grid reference unambiguously identifies a specific location on the earth’s surface. For these reasons, ChEBI is widely used as a small-molecule reference database by a number of leading global bioinformatics resources such as Gene Ontology [6], UniProt [7], Rhea [8], MetaboLights [9], and Reactome [10], among many others. ChEBI is curated by chemistry experts and provides a reliable, non-redundant collection of chemical entities and related metadata such as detailed chemical structure, structural identifiers (InChI, InChIKey, and SMILES), synonyms, molecular formula, net charge, molecular mass, monoisotopic mass, links to external databases, species data for natural products, and relevant citations [5].

Furthermore, and uniquely, ChEBI also contains an extensive ontology that enables the relationships between chemical entities to be defined on the basis of their shared chemical structural features together with their biological/chemical roles. The scope of ChEBI is broad, the database encompasses not only biochemical compounds but also pharmaceuticals, agrochemicals, mixtures, atoms, and subatomic particles. As a general rule, large macromolecules such as proteins and nucleic acids are excluded from ChEBI. All of the data in ChEBI is freely available and downloadable without restriction [5].

ChEBI was first publicly released in July 2004 [5], comprising a public web browser interface, publicly accessible SOAP-based web services for programmatic access, an in-house curator tool for ChEBI curators to annotate entries, a registration-based submission tool for external users to submit compounds to ChEBI, and downloadable data files in various formats [11]. Over the intervening 21 years, the software infrastructure of ChEBI has evolved but it remains dependent upon an outdated operating system, database infrastructure, and software versions, which made it difficult and costly to maintain. In addition, the data integration and release system were not fully automated, meaning that ChEBI required significant daily manual intervention to keep the resource up and running leaving little time for improvements and additions to the platform. Having secured funding from UKRI/BBSRC, we therefore embarked on the critical task of re-engineering a robust platform for ChEBI that is sustainable for the future.

## New infrastructure

### Database

ChEBI was originally designed as a relational database implemented in an Oracle database server [5]. The database schema contained redundant tables and overly complex domain logic. All of the existing data from the commercial Oracle-based system were migrated to a more streamlined schema within PostgreSQL, offering an open-source, cost-effective, and highly extensible solution.

### Database schema changes

Each data item in ChEBI (e.g. synonyms, cross-references, registry numbers, citations) is fully traceable and explicitly referenced to its original source. To simplify the data model, we decided to refactor the source table that contains the source information. In the legacy model, string fields were connected across various tables to display the full details of a particular source. The majority of the business logic was in the codebase, making it difficult for curators to update names and URLs of the data sources. In the new schema, all source data is in one central table and connected to other tables via surrogate keys offering a more dynamic control over the data. ChEBI curators can now easily add new data sources and/or update names and URLs of existing data sources in the database. Each data source has been specifically categorized based on the data it provides. For example, a curator can add a new database as a source that provides synonyms and CAS numbers, and these changes will be automatically deployed. The latest database schema for ChEBI is available (Supplementary Fig. S1).

### Kubernetes deployment

The legacy ChEBI web interface and web services were deployed on virtual machines. All ChEBI services (i.e. public interface, submission portal, and web services) are now deployed as containers into a Kubernetes cluster (https://kubernetes.io/), thereby allowing them to operate independently of the underlying hardware architecture, resulting in improved scalability, reliability, and maximum uptime.

### New website

**Figure 1.**
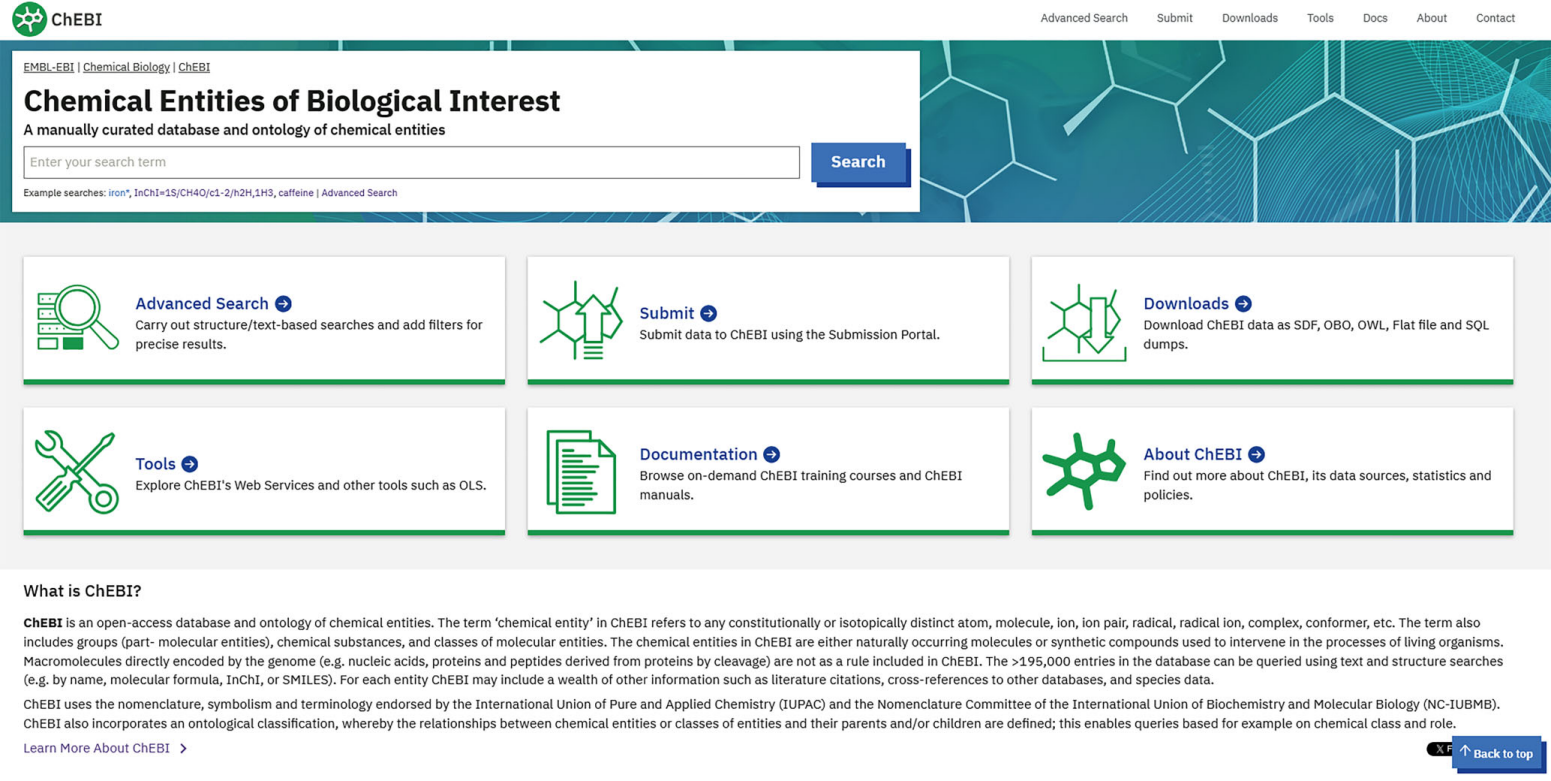
The ChEBI web interface has been completely redesigned. A large search bar at the top left allows users to search for data in the database. The main homepage contains six customized cards leading to easy navigation to data of interest. The cards can also be accessed via the tabs on the top right. Underneath the cards, a short description of ChEBI is provided.

In 2024, 856K users accessed the information contained within ChEBI via its public website available at https://www.ebi.ac.uk/chebi/. The legacy ChEBI web interface provided multiple facilities for searching (text and chemical structure-based), browsing, and filtering. Results were displayed via different tabs to enable easy navigation to data of interest. ChEBI’s web interface had evolved over the years in response to user feedback [12]. However, its non-responsive design, text-heavy content, and web design (colour scheme, font, and layout) were now significantly outdated. We therefore redesigned and developed a new web interface for ChEBI through an iterative process in collaboration with the user experience (UX) team at EMBL-EBI. A new logo, hero image, and several icons were designed for the new homepage to consolidate the available functionality (Fig. 1). The redeveloped website uses a contemporary frontend framework (Visual Framework v2.5—https://stable.visual-framework.dev/) that provides prebuilt UX design patterns, which helps ensure EMBL-EBI brand consistency across the institute’s data resources, and enables the use of modern web design best practices such as responsive design. It is created as a separate frontend application and integrated with the backend via ChEBI’s REpresentational State Transfer (REST) API unlike the legacy model in which the pages were rendered server-side in the monolithic application. Overall, the new website continues to provide the same general capabilities as the legacy web interface. The new design allows greater adaptability to future modifications.

### Improvements to website

**Figure 2.**
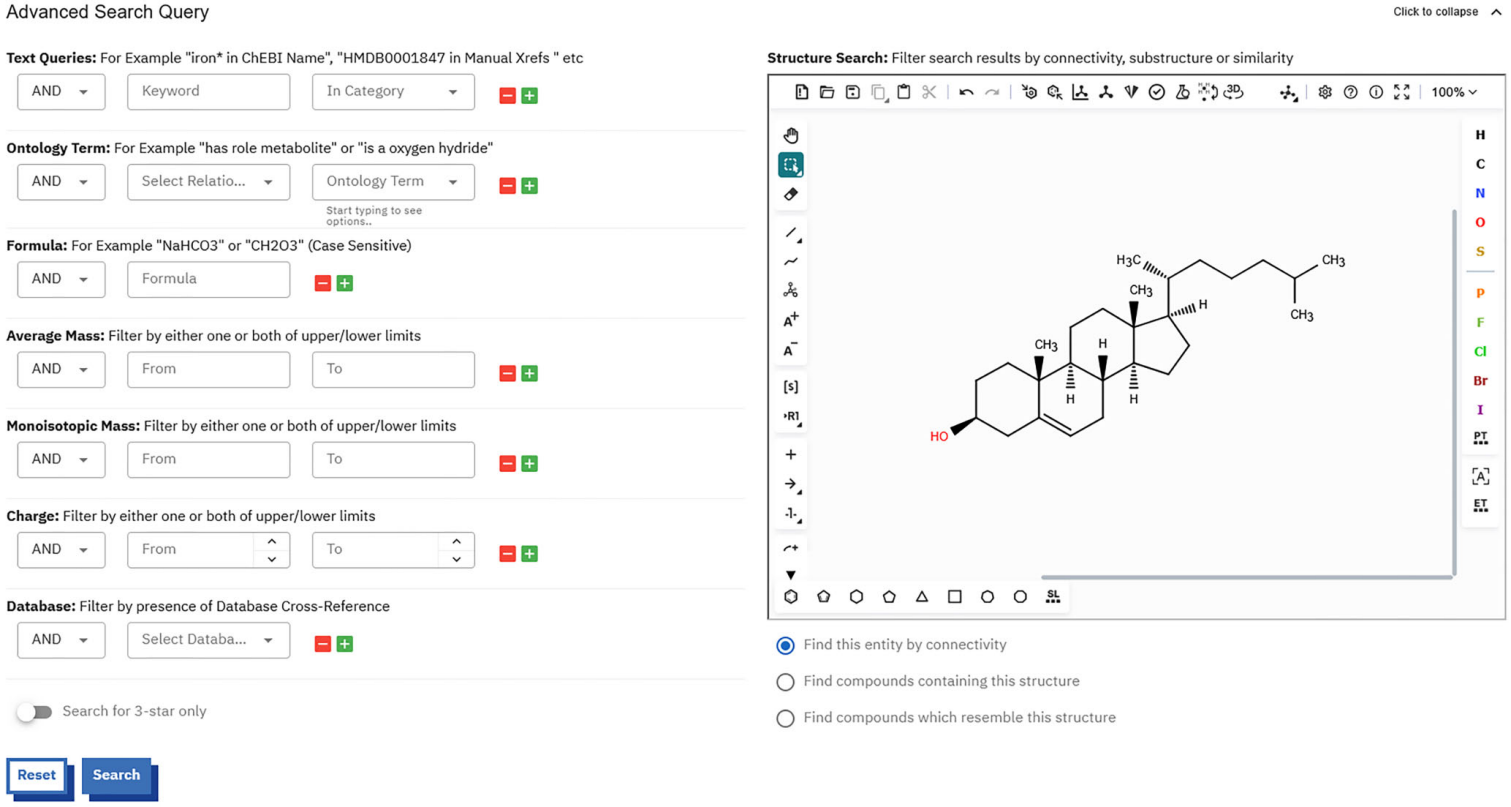
A screenshot of the new ChEBI advanced search interface. Text-based searches and filtering options provided on the left and structure editor (Ketcher) found on the right.

ChEBI’s legacy search interface contained a text search as well as a structure search. The advanced search enabled users to search all of the public data that ChEBI offered or search by category (e.g. ChEBI ID, names, database links, formula, SMILES, and InChI/InChIKey). Mass, monoisotopic mass, and charge could be searched within ranges. Users could also search for entities with a specific formula and searches could be filtered by database of origin; for example, one could search for entities that have cross-reference links to the PDBe [13] or ChEMBL [14] databases. Users could also filter by ChEBI ontology terms allowing the retrieval of all children of a specific entity based on the relationship given. All of the searches could be combined using logical operators AND, OR, and BUT NOT. Search results could be exported in several file formats [11]. We have redeveloped the advanced search facility (Fig. 2) so that it continues to provide the same general capabilities.

#### Text searching

The legacy ChEBI used Apache Lucene, a file-based search engine for text searching [11]. This had the major drawback of not supporting concurrency, which prevented separate ChEBI applications (e.g. curator tool and public website) from accessing the same index at the same time. We have replaced Lucene with the widely used Elasticsearch engine (https://www.elastic.co/elasticsearch), a distributed, RESTful search and analytics engine, providing multiple advantages such as concurrent access for multiple applications, improved scalability, faster search performance, and powerful query capabilities.

#### Chemical structure searching

The new ChEBI provides three options: Connectivity search (‘find this entity by connectivity’), substructure search (‘find compounds containing this structure’), and similarity search (‘find compounds which resemble this structure’). For structure searching, ChEBI continues to provide an open-source framework for chemistry and has moved away from OrChem (https://orchem.sourceforge.net/) to cartridges based on RDKit, a leading open-source cheminformatics toolkit. For structure connectivity searches, we use a computational efficient strategy by executing an exact text match of the first hash block of the InChIKey, enabling rapid searching of compounds with identical connectivity. For substructure searches, ChEBI uses the RDKit module rdkit.Chem.rdSubstructLibrary (https://www.rdkit.org/docs/source/rdkit.Chem.rdSubstructLibrary.html) to improve search efficiency. For compound similarity searches, ChEBI utilizes FPSim2 (https://chembl.github.io/FPSim2/), a NumPy-centric Python/C++ RDKit-based package that is widely used by other chemistry resources at EMBL-EBI including ChEMBL [14] and SureChEMBL [15].

#### Search results

To maintain computational efficiency, a limit is set on the number of results returned by overly broad queries; substructure searches in particular can sometimes yield excessively large result sets. This consideration is especially relevant for the ChEBI web services that operate over the internet and are accessed by a global user base. Consequently, all searches performed on the web interface and web services are restricted to a maximum of 30 000 results. This constraint reflects the fact that queries are processed in memory on the ChEBI servers, after which the results are integrated via document identifiers with data stored in Elasticsearch and/or PostgreSQL.

#### Structural properties

Formula, net charge, average mass, monoisotopic mass, images, and SMILES are generated by libRDChEBI (https://github.com/chembl/libRDChEBI/), a Python wrapper that uses RDKit functionality. InChI and InChIKey are directly retrieved from RDKit via rdkit.Chem.rdinchi module (https://www.rdkit.org/docs/source/rdkit.Chem.rdinchi.html). ChEBI now provides Web3 Unique Representation of Carbohydrate Structures (WURCS) notation [16] for all ChEBI entries that fall under the carbohydrate (CHEBI:16646) ontology class and these are generated using the MolWURCS Java library (https://gitlab.com/glycoinfo/molwurcs). Users can search for WURCS on the web interface (via the search bar or by selecting WURCS from the drop-down menu of the text queries section of the Advanced Search page). WURCS are also included in the downloadable files.

#### Manual cross-references

ChEBI provides database cross-references to several data sources directly. A major limitation of this approach is that quite often a data source may change its URL or become merged with another source or even become deprecated. Instead of manually adding the URL that links to another data source, we now use Bioregistry [17] to generate the URL given the database identifier, so offering a standardized and a more consistent approach.

#### UniProt name

UniProt Names in ChEBI are assigned by UniProt curators. They are extensively used in UniProt [7] and Rhea [8] to describe ChEBI compounds. They were previously displayed in the synonyms section of a ChEBI entry. However, these names are sometimes not strictly correct according to chemical naming conventions. For example, the UniProt Name ‘a fatty acid’ has been assigned to fatty acid anion (CHEBI:28868) instead of fatty acid (CHEBI:35366). To avoid confusion, we decided to migrate all UniProt names from the synonyms section to a new section called UniProt name.

#### Species of metabolite

For entities that have been detected in or isolated from living organisms, the species name, link to an appropriate taxonomy and component (stem, root, blood, etc.) where the entity is found, is provided, together with a relevant citation. After discussions with the MetaboLights [9] team, the previous ‘Metabolite of Species’ section was renamed to ‘Species of Metabolite’ since the primary focus of this section is on the different types of species rather than the metabolite itself. A minor refactoring of the species table was carried out. The size of the species table has been extended from two columns to four to make the data more visible. The first column provides the species name with a relevant taxonomy accession number, the second column provides the component name with a relevant ontology accession number, the third column provides the source of the data, and the fourth column provides submitter/curator comments about the data.

#### Ontology visualization

**Figure 3.**
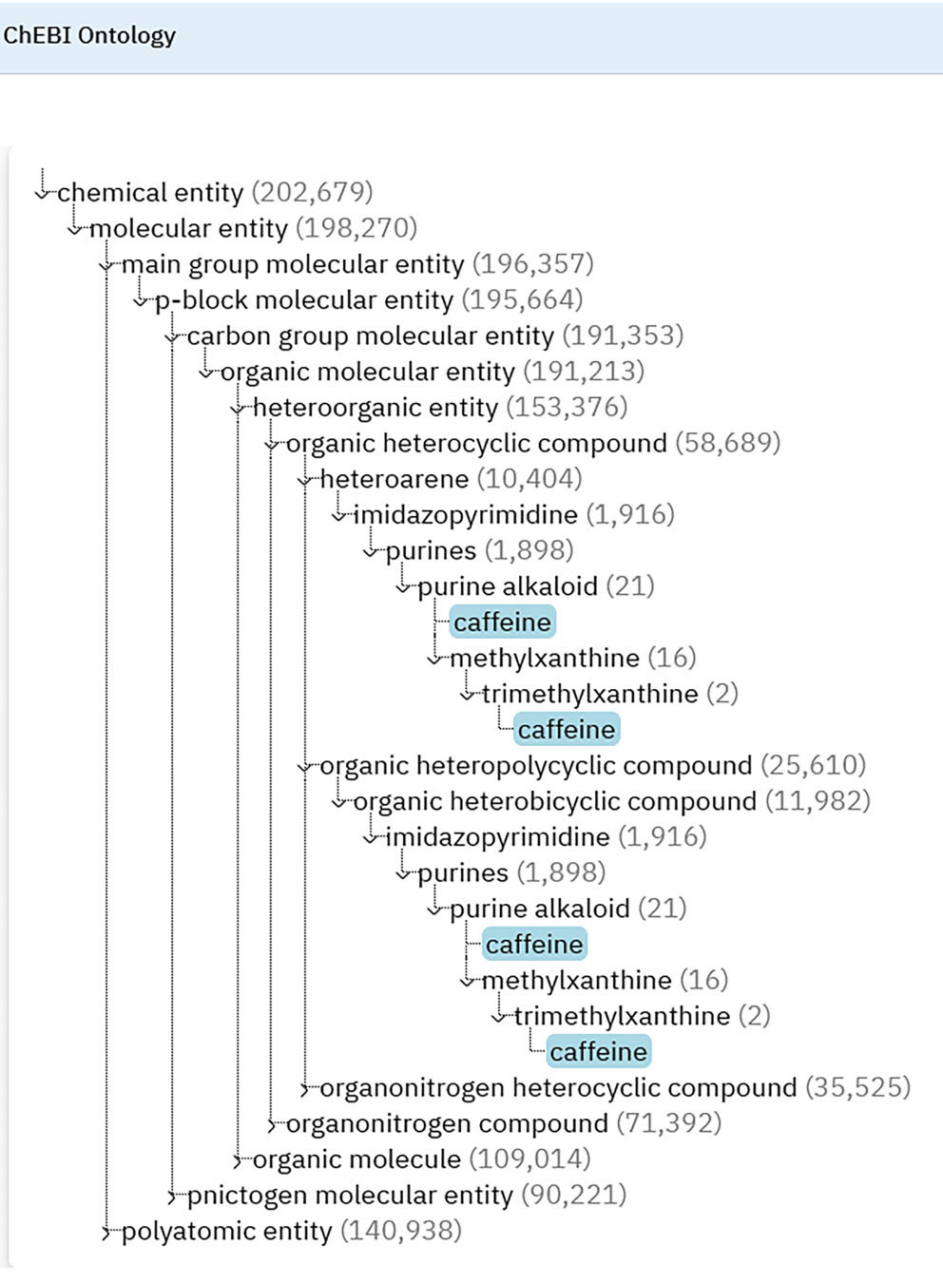
The tree view shows the hierarchical structure of a ChEBI entity, displaying its position within the overall classification from the ChEBI root class down to the specified entity. Users can expand or collapse the hierarchy nodes by clicking on the arrow next to each term. A number is displayed in brackets next to each term that shows the total number of members in each class. The widget is interactive and offers a clear and intuitive way to explore the hierarchical structure of ChEBI entries. The view enables users to effectively navigate through different levels of the hierarchy and understand relationships between ChEBI entities.

**Figure 4.**
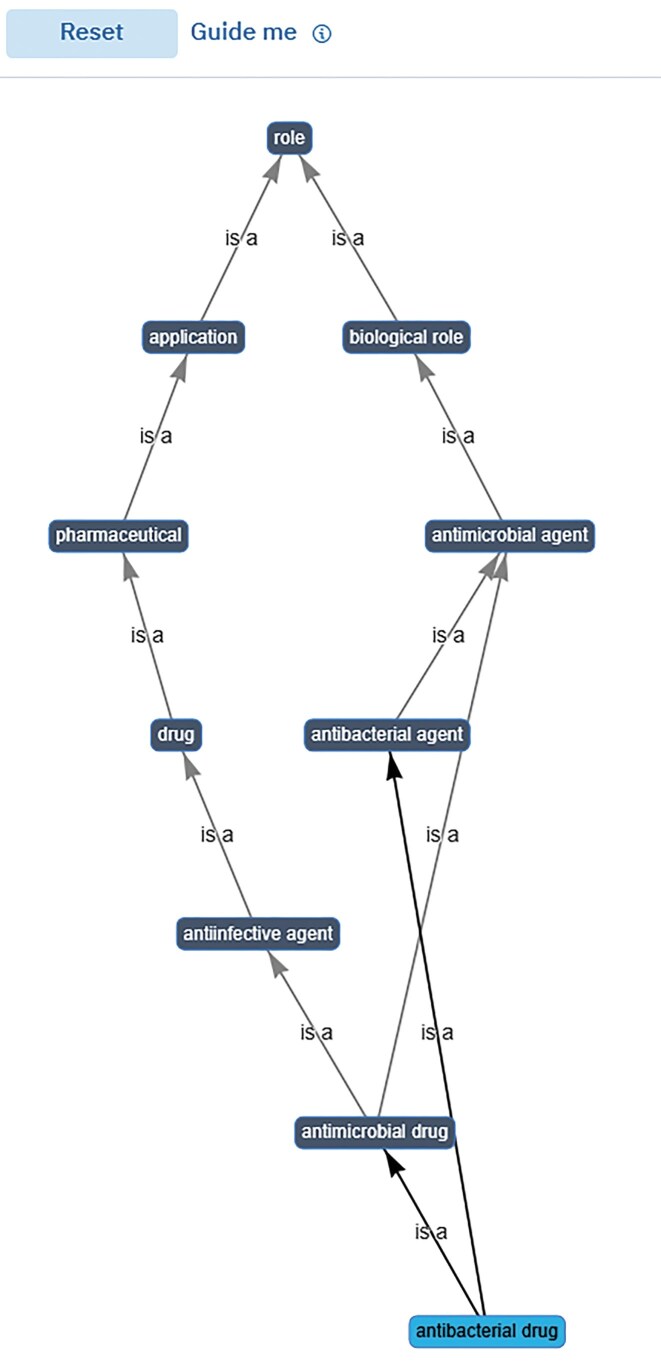
The graph view shows the ChEBI entities as nodes and their relationships as edges, enabling users to interact by zooming, panning, and dragging elements. Double-clicking a term in the graph leads you to the entry page of that term, providing a seamless exploration of the entry. The root walk and hierarchy modes offer optional views, where users can trace paths from any selected entity to the root nodes of the ontology, enhancing contextual understanding. The Guide me functionality provides a help popover with usage tips. The reset button restores the graph to its initial state for ease of navigation.

In the legacy website, the ontology data for each entity was displayed across two tabs (Main display and ChEBI ontology), meaning that data related to roles and applications were duplicated in two separate places on the web interface. In the new design, most of the ontology information has been removed from the ontology tab and moved to the main display page of an entry. Users can now visualize the ChEBI ontology in text, tree, and graph views by clicking on the appropriate tabs within the ontology section. In the text view, users can see all of the ontology relationships assigned to an entry (including outgoing/incoming relationships). In the tree view, the ‘is a’ relationships are shown in a fully interactive hierarchical display, illustrated in Fig. 3. The tree view is generated using a JavaScript-based widget developed by Terminology Services 4 NFDI (TS4NFDI) (https://base4nfdi.de/projects/ts4nfdi), which displays the hierarchical structure of a ChEBI entry, specifically the upper-level classes of an entity within the ChEBI ontology. The widget visualizes the entity’s position within the hierarchy, starting from the root class of the ontology and expanding down to the specified entry. Users interact with the hierarchy by expanding or collapsing nodes to navigate through related classes or entities. In collaboration with TS4NFDI, we also developed a customized graph-view widget for ChEBI (Fig. 4). The source code of the widgets is available at https://github.com/ts4nfdi/terminology-service-suite?tab=readme-ov-file. In the graph view, the entity of interest appears at the bottom of the graph linked via ‘is a’ relationships to the root class, which can be a chemical entity, role, or subatomic particle in a hierarchical manner. Both widgets have been built to use data directly from the Ontology Lookup Service data model [18] instead of ChEBI to generate the views.

### Web services

The ChEBI web services provide programmatic access to all information in the ChEBI database so that users can integrate the data into their applications. The database was originally using Simple Object Access Protocol (SOAP), an XML-based protocol to deliver web services over Hypertext Transfer Protocol (HTTP) [5]. We have replaced this with the preferred REST, which is lightweight and more flexible, and provides superior performance. REST has been widely adopted by the scientific community to access data programmatically and permits a greater variety of data formats, allowing users to access data in ways that are more compatible with their requirements than is possible with SOAP.

The new REST web services were written in Python with the widely used Django 5.0 Python-based web framework (https://www.djangoproject.com/) and can be explored via an interactive interface with the aid of Swagger UI at https://www.ebi.ac.uk/chebi/backend/api/docs/. This interface simplifies the navigation and testing of endpoints and markedly improves the APIs accessibility. The functionality provided by the legacy ChEBI web services has been reproduced in the new implementation. The API endpoints allow users to perform keyword searches, conduct advanced searches within ChEBI, and retrieve detailed information about specific ChEBI entries. The input parameters and output responses are formatted in JSON.

### Downloads

**Figure 5.**
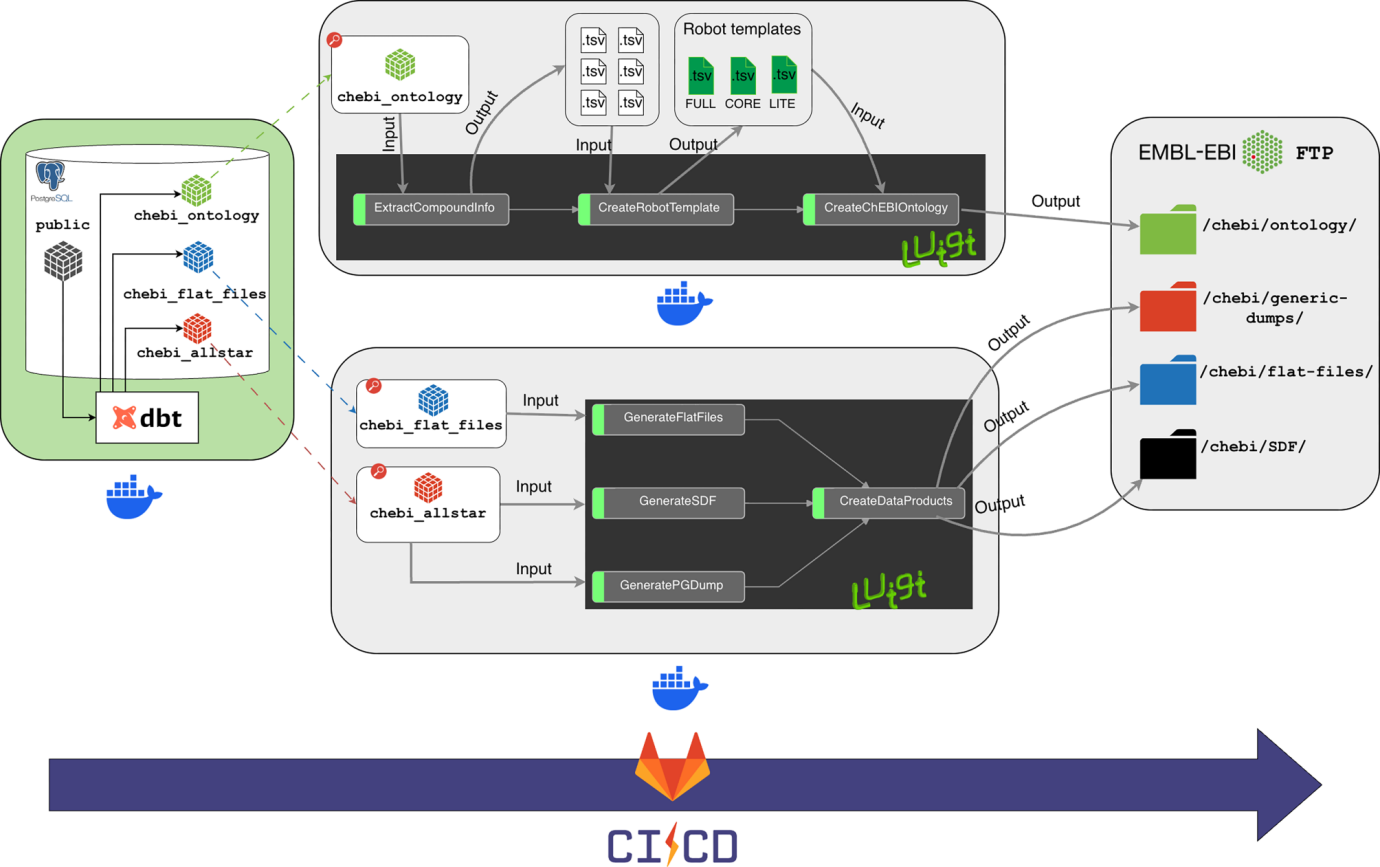
Downloadable files are generated using three separate data pipelines. The first pipeline generates the database models for each data product using dbt. The second pipeline generates the ontology files (OWL, OBO, and JSON formats). The third pipeline generates the PostgreSQL dump, flat files, and SDF files. The three pipelines are orchestrated by GitLab CI/CD.

ChEBI exports data in several file formats each month including TSV, SDF, OBO, and OWL and also provides database dumps for different user communities. We have developed a new data file generation process (Fig. 5) that ensures ChEBI’s data exporting pipelines are maintainable, scalable, and testable during the monthly release cycle. Additional data download formats will be made available in due course according to user feedback.

#### Data transformation

We used dbt (https://www.getdbt.com/), a development framework to create a new data pipeline that transforms the raw data in the PostgreSQL database into reliable data products. The use of dbt allowed us to decouple the data transformation logic into three separate sets of dbt models depending on the type of data product: Ontology, Flat files (TSV), SDF files, and PostgreSQL dump. ChEBI’s public database schema was used as the source of truth to build the individual dbt models. Each dbt model is an .SQL file with a SELECT sentence, enabling the selection of specific columns (data) from the public database schema object (table/view). The repository that contains the dbt model generation code is available at https://gitlab.ebi.ac.uk/chembl/chebi/chebi-2.0/chebi-data-management.

#### New data workflow

We developed two additional data pipelines with custom logic using Luigi (https://luigi.readthedocs.io/en/stable/), an open-source Python framework that can be run in batch mode and offers enhanced workflow management. The first pipeline (https://gitlab.ebi.ac.uk/chembl/chebi/chebi-2.0/chebi-ontology-generator) generates the ChEBI ontology files using the ontology dbt models and ROBOT templates (https://robot.obolibrary.org/template). The second pipeline (https://gitlab.ebi.ac.uk/chembl/chebi/chebi-2.0/chebi-dumps) generates the TSV, SDF, and the PostgreSQL dump using the applicable dbt models.

#### Quality control

The new data workflow makes extensive use of the OBO tool, ROBOT [19], to check for inconsistencies in the ontology files before releasing them (see https://robot.obolibrary.org/report.html). The legacy ChEBI ontology files had no external QC checks in place resulting in 1308 errors according to ROBOT. The majority of the errors were primarily caused by duplicate textual definitions and missing core ontology metadata, such as the ontology’s title, description, and license. We resolved this by removing the duplicate definitions from the new ontology files and adding the required metadata. As a result, the new ontology files contain zero errors and now conform to OBO foundry principles.

#### Pipeline execution

The entire data file generation process is run in a Docker container (https://www.docker.com/). The data pipelines are fully automated and orchestrated by Gitlab CI/CD (https://about.gitlab.com/topics/ci-cd/). The system generates the data files in the correct sequence, performs the unit tests that enables the identification of failures in the pipeline without searching through the job logs, carries out the data tests to detect changes to the data, and finally releases the data files into the EBI FTP site (https://ftp.ebi.ac.uk/pub/databases/chebi/). Each ChEBI release has a GitLab tag assigned, giving transparent version control over the ChEBI data pipelines. Every change is committed and traceable, so the team knows exactly what change has been made, who did it, and when it was done, thereby allowing easier monitoring of the ChEBI release versions. The new streamlined process can generate all of the data files within 36 min, as compared to the legacy system which took 1 h 56 min.

#### New file formats

ChEBI continues to be provided in file format to enable key communities to integrate ChEBI data with their systems, with the exception that Oracle database dumps have been replaced by PostgreSQL dumps. Leveraging the new ontology generation process, we have also included an additional OBO JSON Graph format (https://github.com/geneontology/obographs) for all ontology variants: LITE, CORE, and FULL. In addition to the regular monthly releases, the ontology file in JSON format will be updated on a nightly basis along with the OWL and OBO files.

#### Improvements to ontology data files

We have standardized the data within the OBO and OWL files. The legacy files used a mixture of different prefixes for annotation and object properties, an unintended consequence of the previous ontology generation process where the OWL file was generated from the OBO file. For example, http://purl.obolibrary.org/obo/chebi# was used for the annotation properties and http://purl.obolibrary.org/obo/chebi/ was used for the object properties. We decided to replace these with a single prefix to represent both annotation properties and object properties: http://purl.obolibrary.org/obo/chebi/. The legacy OWL file also used a custom hashed prefix for annotation properties, e.g. http://purl.obolibrary.org/obo/chebi#mass was used to represent average mass. We have standardized this and now use CHEMROF (https://w3id.org/chemrof/) prefixes for the chemical data (Table 1). The legacy files used a custom slashed prefix for ontology relationships, e.g. http://purl.obolibrary.org/obo/chebi/has_functional_parent was used to represent the ‘has functional parent’ ontology relationship. We now use the OBO Relation Ontology (https://github.com/oborel/obo-relations) to model relationships between chemical entities, thereby increasing the semantic interoperability of the ontology (Table 2). The legacy files did not have the correct XML data types for annotation properties; charge, mass, and monoisotopic mass in the files were represented as strings. Charge is now represented as an integer, and mass and monoisotopic mass as decimals. The cross-reference prefixes in the legacy files were not homogenized. For example, one could find two different prefixes for the same database source within the ontology file (GitHub issue: https://github.com/ebi-chebi/ChEBI/issues/4489). To standardize the prefixes, we decided to use Bioregistry [17] as our main prefix repository and the service also resolves Compact Uniform Resource Identifiers in ChEBI.

**Table 1.** New annotation properties in the OWL file and their equivalence in the legacy OWL file

Old CEBI OWL file	New ChEBI OWL file
chebi:charge	chemrof:charge
chebi:formula	chemrof:generalized_empirical_formula
chebi:inchikey	chemrof:inchi_key_string
chebi:inchi	chemrof:inchi_string
chebi:monoisotopicmass	chemrof:monoisotopic_mass
chebi:mass	chemrof:mass
chebi:smiles	chemrof:smiles_string
wurcs not provided	chemrof:wurcs_representation

**Table 2.** The new ontology files are using RO identifiers to model relationships between entities

Old ChEBI	New ChEBI
has_functional_parent_parent	RO:0018038
has_parent_hydride	RO:0018040
is_conjugate_acid_of	RO:0018034
is_conjugate_base_of	RO:0018033
is_enantiomer_of	RO:0018039
is_substituent_group_from	RO:0018037
is_tautomer_of	RO:0018036
has_parent_hydride	RO:0018040

The old files were using chemistry-specific relationships.

### Submission portal

The growth of ChEBI has been historically driven by its user community, who have contributed >101K entries to date. A key advantage of ChEBI is that it is manually curated by experts. Users from the bioscience community typically request the addition of new chemical entities to the ChEBI team via the submission tool [11] or via ChEBI’s GitHub issue tracker (https://github.com/ebi-chebi/ChEBI). The chemical structure of each entry is then drawn by an expert ChEBI curator, classified within the ontology, and assigned multiple annotations including (where relevant) metabolite species information, database cross-references, synonyms, and literature citations. Once the new entry is complete, it is made available immediately via the website and the requesting party is informed that the request has been fulfilled. The downloadable files are subsequently updated on a monthly basis with the new entry. ChEBI’s legacy infrastructure had two separate tools to submit and process new entries: a submission tool that enabled external users to submit new structures along with their associated metadata into ChEBI and an internal curator tool that was used by the ChEBI curators to annotate the entries that were being submitted to ChEBI. We have developed a new simple graphical user interface (GUI) for both internal curators and external submitters that we call the ‘Submission Portal’ (available online at https://www.ebi.ac.uk/chebi/backend/submit/). This single platform offers differential read/write permissions giving fine-gained control over which data can be modified by users, delivering lower future maintenance overheads and making it easier to incorporate future enhancements.

#### Backend

**Figure 6.**
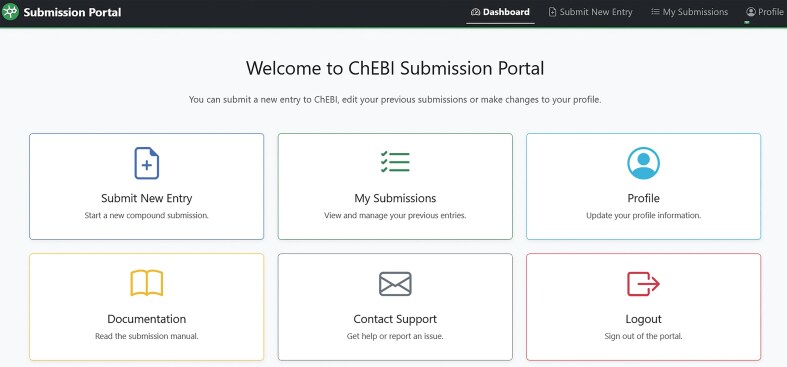
Submission Portal UI showing a series of cards; the Submit New Entry card allows users to submit new entries to ChEBI; the My Submissions card enables users to view their submitted entries and edit them. The Profile card allows users to change their profile settings. The Documentation card links to the Submission Portal manual. The contact support card allows users to send a message directly to the ChEBI team for help. The logout card will allow users to log out of the system.

The codebase of the Submission Portal was written in Python and developed using Django Python web framework (https://github.com/django/django). This framework is also employed by ChEMBL [14] allowing easier maintenance and sustainability. The user interface (UI) was developed using Bootstrap 5 (https://getbootstrap.com), providing rapid prototyping and optimal user experience across different devices (Fig. 6).

#### Migration of existing user accounts

Nine hundred twenty-five user accounts were registered with ChEBI’s submission tool in the legacy Oracle database. The old registration system allowed users to set up multiple accounts using different usernames, resulting in 163 duplicate user accounts. The inactive duplicate user accounts have been removed and the remaining 762 unique user accounts transitioned into the new PostgreSQL system.

#### Registration

A new registration process has been developed for access to the Submission Portal that enforces one unique user account per email address in contrast to the legacy registration system. New users must apply for an account using their e-mail address, providing their first name, last name, the name of the organization they work for, and their organization address. In the legacy system, submitters had the option to provide a public name that appeared in the submitter section of the submitted entry. In the new system, while a submitter’s first and last name will be used as default, they have the option to provide a different public name if they wish or remain anonymous. During the registration process, the user’s email address is verified, and their credentials are checked by the ChEBI team using ChEBI’s Django admin site to prevent the authorization of fictitious users. Once a user has been authorized, they will be sent another email to create a new password and begin submissions.

#### Submission workflow

In the legacy submission tool, submitters provided data across multiple tabs. We have simplified the submission process by allowing submitters to provide all the relevant data on a single page (Supplementary Fig. S2), thereby increasing submission efficiency. Once all of the relevant data is provided, a ChEBI ID will be generated immediately after clicking the Submit Entry button. Submitters can view the data they have provided and can edit the data if required. Submitters can also view their submitted entry on the public website.

#### Requirements for a new submission

The minimum data that is required for a ChEBI submission remains the same [11]: a unique ChEBI name within the database, a primary classification within the ontology, and either a chemical structure or a text definition.

#### Checks

Basic checks and validations have been built into the new Submission Portal. For the detection of duplicate chemical structures, we have implemented RDKit module rdkit.Chem.RegistrationHash (https://www.rdkit.org/docs/source/rdkit.Chem.RegistrationHash.html), initially introduced by Schrodinger. For the detection of duplicate chemical names, a traffic light warning system has been implemented. If a duplicate ChEBI name is provided, a red warning will be issued preventing the user from submitting the entry since each ChEBI name must be unique. If the submitted ChEBI name is an IUPAC name, synonym, International Nonproprietary Name, or brand name of an existing entry, a yellow warning will be issued to the user to inform them that an entry already exists in the database with that name. However, they can still submit the entry by ignoring the warning. If the ChEBI name is unique, and not found in any other entry, a green approval will be issued informing the user that the ChEBI name is unique and that they may proceed with their submission. To ensure validity of ontology relationships, we have implemented most of the existing ontology checks that have been built into ChEBI over the years. Specifically, duplicate ontology relationships are not allowed; an entry must have at least one ‘is a’ relationship; if an entry has unclassified relationship then its not allowed to be checked; if a relation is cyclic then it should have a complementary reverse relationship in the opposite direction (i.e. if Entry A is tautomer of Entry B, the reverse relationship also holds true, Entry B is tautomer of Entry A, etc.).

#### Saving entries

To simplify the data model, we decided to remove submission IDs for each submission. Users are no longer able to save partially completed entries in the Submission Portal and work on those entries at a later date/time.

#### Editing submissions

Users have the ability to view their own submitted entries and can continue to edit their own submissions until the entry has been checked by the ChEBI team.

#### Systematic names

Users could previously provide systematic names with their entries, however most users could not differentiate between a systematic name and a synonym, and the category was rarely used. To simplify the data model, the systematic name category has been removed from the database and all existing systematic names have been recategorized as synonyms.

#### Data sources

New data sources have been added to the Submission Portal, e.g. MeSH [20] has been added as a new data source for manual cross-references. CAS Common Chemistry [21] has been added as a new data source for synonyms and CAS registry numbers. The source DrugBank [22] has now been split into the separate sources, DrugBank, DrugBank Salts, and DrugBank Metabolite enabling accurate cross-references to the specific categories of drugs, salts, and metabolites.

#### Strain data

Strain accession numbers can now be provided as an option in the Submission Portal along with the strain name. For example, a chemical entity may be identified or detected in BALB/c, an inbred albino mouse strain. The species name will be *Mus musculus* (NCBI:txid10090) but the exact strain name will be BALB/c whose strain accession number will be EFO:0000602 (http://www.ebi.ac.uk/efo/EFO_0000602).

#### Data cleanup

The legacy submission tool allowed submitters to provide species names without relevant taxonomy identifiers, and component names were being added into the species name section. We decided to carry out a major cleanup of the species data table to improve data quality, replacing non-standard sources with existing sources and linking each species record to an appropriate taxonomy identifier. ChEBI now provides six standard sources for species accessions and these include Index Fungorum [23], Integrated Taxonomic Information System [24], International Plants Names Index [25], MycoBank [26], National Center for Biotechnology Information taxonomy [27], and World Register of Marine Species [28]. If a specific species accession is not found in these existing databases then a new data source can be added to the database upon request.

### Data content

ChEBI currently includes 201 471 entries of which 61 961 entries have been fully curated by the ChEBI team (as of release 244). Natural products are of significant interest in metabolomics and drug discovery. Since the last update in NAR in 2016 [29], some large data depositions have been made to ChEBI by our partner database MetaboLights [9]. A subset of metabolites (>16 000) from the Metabolomics Workbench RefMet database [30] was deposited into ChEBI in 2021, followed by >31 000 natural products from the Natural Products Atlas database [31] in 2024. The latter included metabolite species information and source information, e.g. the publication that reported the identification of the particular metabolite in a given species. The total number of entries classified as metabolites in the ChEBI ontology has now surpassed 26 100. In 2020, a collaboration with the GlyGen database resulted in the deposition of >10 500 glycans into ChEBI [32]. Additional cross-references to the following databases have been added, including to GlyGen [33], GlyTouCan [34], FooDB [35], MetaCyc [36], BCPC compendium of pesticide common names (formerly known as Alan Wood’s pesticides database) [37], the University of Hertfordshire databases that includes Pesticides Properties DataBase, Veterinary Substances DataBase, and BioPesticides DataBase [38]. ChEBI now provides >439 000 manual cross-references to other data resources and we expect this figure to grow in the coming years. Links to deprecated sources (e.g. Enzyme Portal [39] and IntEnz [40]) have been removed from ChEBI.

### User engagement

The new infrastructure provided clear opportunities to seek input from the wider user community. In November 2024, we hosted a hybrid workshop at EMBL-EBI for key resource groups, to update on progress, receive feedback, and to solicit further input. These interactions were central to shaping the future development and enhancement of ChEBI. A key topic of discussion at this workshop focused on the complexity of ChEBI’s ontology stemming from, among other issues, multiple compound protonation states and tautomers. A proposal was made for a simpler, biology-focused ontology and this is a goal of future versions now that the underlying codebase has been updated. News and information on the project and its progress were shared with ChEBI’s wider community via multiple mechanisms including the public website, mailing lists, blog posts (https://chembl.blogspot.com/), and social media (https://x.com/chebit). Some of our users, particularly from the Rhea team and OBO community, provided valuable feedback leading to improvements to the downloadable files, and others acted as beta testers during the project. We are grateful to all users and collaborators who have helped with the development of the new system.

## Summary

For the past 21+ years, ChEBI has provided the scientific community with a ‘gold standard’ in data annotation for chemical entities. In this update, we have described ChEBI’s redevelopment that has been made possible by recent advances in software development techniques. In 2017, ChEBI was designated an ELIXIR core data resource (https://elixir-europe.org/platforms/data/core-data-resources) and subsequently in 2022 as a Global Core Biodata Resource (https://globalbiodata.org/) in recognition of its fundamental importance to the wider life-science community. Despite ChEBI’s importance, funding of projects of this kind remains challenging and very limited. We have overcome this hurdle and have delivered a new infrastructure platform that is critical to enabling ChEBI to continue to fulfil the vital role it plays in the global bioinformatics community. The new infrastructure provides all of the main capabilities of the legacy system and continues to be developed. We will now focus our efforts on the redevelopment of the curation interface by developing new facilities within the Submission Portal to enable ChEBI curators to edit any data in the database. Our aim is to deliver a tool that facilitates the creation and deposition of new entries, in a way that makes the overall process as efficient as possible, for example by incorporating automation. We also intend to make these capabilities more widely accessible, for example to key collaborators, to enable community curation. Existing features that are currently missing such as automatic cross-references will be added gradually over time. The new infrastructure will also enable ChEBI to actively move to a different operating model in which depositors wishing to make significant depositions into ChEBI would be expected to provide the (human) curator resources to cover their contributions. EMBL-EBI would then provide curator and software developer resources sufficient to meet small-scale requests from the user community and to maintain the underlying infrastructure. In the near future, we plan to organize a programme of virtual webinars and online training courses for the ChEBI user community. Such virtual training has proved very popular during the recent covid pandemic, but even in normal times represents a very efficient way to reach a wide, geographically diverse audience in an eco-friendlier way.

## Supplementary Material

gkaf1271_Supplemental_File

## Data Availability

All data in ChEBI are freely accessible and available to anyone under the Creative Commons CC BY 4.0 License. Furthermore, each data item comes with full provenance and explicitly referenced to its original source. Besides web access and web services, the entire ChEBI data are provided in several different formats on our FTP server (https://ftp.ebi.ac.uk/pub/databases/chebi/). The ChEBI source code is publicly accessible and can be downloaded from its GitLab repository (https://gitlab.ebi.ac.uk/chembl/chebi/chebi-2.0/).
