# Supplementary material for: ChEBI: re-engineered for a sustainable future
Source: Nucleic Acids Res. 2025 Nov 28;54(D1):D1768–78. doi: 10.1093/nar/gkaf1271 (PMC12807787; doi:10.1093/nar/gkaf1271)
Supplement: gkaf1271_Supplemental_File [file gkaf1271_supplemental_file.pdf]

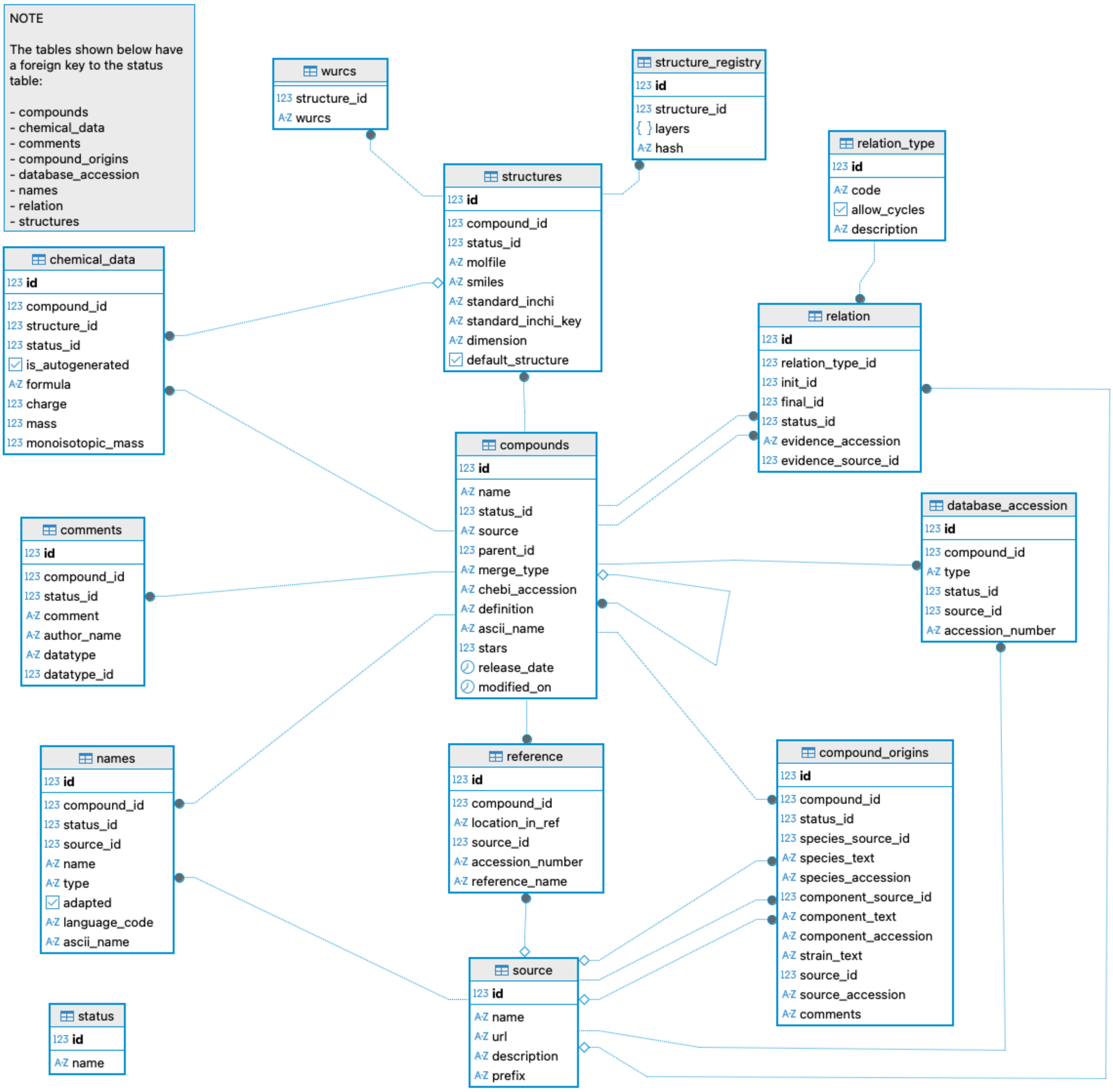

Supplementary Figure 1. The latest database schema for ChEBI.

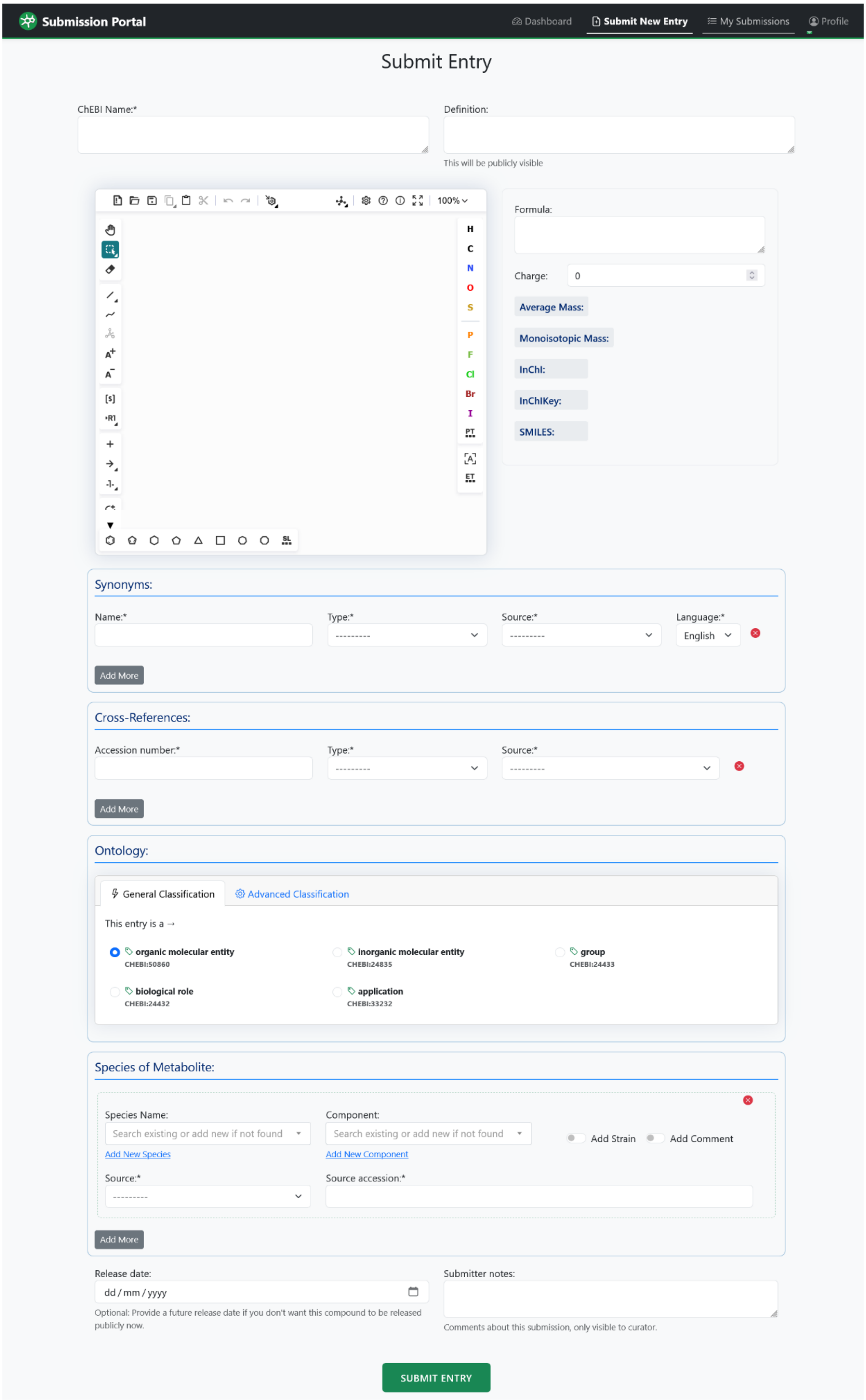

Supplementary Figure 2. Screenshot of the new submission workflow.
